# Editorial Expression of Concern: Tumor necrosis factor-inducible gene 6 protein ameliorates chronic liver damage by promoting autophagy formation in mice

**DOI:** 10.1038/s12276-021-00554-6

**Published:** 2021-02-22

**Authors:** Sihyung Wang, Chanbin Lee, Jieun Kim, Jeongeun Hyun, Minso Lim, Hyuk-Jin Cha, Seh-Hoon Oh, Yung Hyun Choi, Youngmi Jung

**Affiliations:** 1 Department of Integrated Biological Science, College of Natural Science, Pusan National University, Pusan, Korea; 2 Department of Medicine, SogangUniversity, Seoul, Korea; 3 Department of Medicine, Division of Gastroenterology, Duke University, Durham, NC USA; 4 Department of Biochemistry, Dongeui University College of Korean Medicine and Anti-Aging Research Center, Dongeui University, Pusan, Korea; 5 Department of Biological Sciences, College of Natural Science, Pusan National University, Pusan, Korea

**Keywords:** Autocrine motility factor, Adipocytes

The Editor-in-Chief is issuing an editorial Expression of Concern to alert readers that the Committee of Research Integrity of Pusan University has found that Minso Lim, the fifth author listed on this article^1^, did not participate in the study described. Yung Hyun Choi has stated that Minso Lim should be included in the authorship. None of the other authors has responded to emails from the Editor or the Publisher.
